# Valorization of Chinese hickory shell as novel sources for the efficient production of xylooligosaccharides

**DOI:** 10.1186/s13068-021-02076-9

**Published:** 2021-11-27

**Authors:** Zhi-Kun Wang, Caoxing Huang, Jun-Lei Zhong, Yi Wang, Lv Tang, Bing Li, Jian-Jun Sheng, Liang Chen, Shaolong Sun, XiaoJun Shen

**Affiliations:** 1 Zhejiang Provincial Key Laboratory of Carbon Cycling in Forest Ecosystems and Carbon Sequestration, College of Environmental and Resource Sciences, Zhejiang A&F University, Lin’an, 311300 Hangzhou China; 2 State Key Laboratory of Catalysis (SKLC), Dalian National Laboratory for Clean Energy (DNL), Dalian Institute of Chemical Physics (DICP), Chinese Academy of Sciences, Dalian, 116023 China; 3 Jiangsu Co-Innovation Center of Efficient Processing and Utilization of Forest Resources, College of Chemical Engineering, Nanjing Forestry University, Nanjing, 210037 China; 4 College of Natural Resources and Environment, South China Agricultural University, Guangzhou, 510642 Guangdong China; 5 State Key Laboratory of Bio-Fibers and Eco-Textiles, Qingdao University, Qingdao, 266071 Shandong China

**Keywords:** Chinese hickory shell, Hydrothermal pretreatment, XOS, Heteronuclear single quantum coherence (HSQC)

## Abstract

**Abstract:**

Chinese hickory shell, a by-product of the food industry, is still not utilized and urgent to develop sustainable technologies for its valorization. This research focuses on the systematical evaluation of degraded products and xylooligosaccharide production with high yield from the shell via hydrothermal process. The pretreatment was carried out in a bath pressurized reactor at 140–220 °C for 0.5–2 h. The results indicated that the pretreatment condition strongly affected the chemical structures and compositions of the liquid fraction. The maximum yield of XOS (55.3 wt%) with limitation of by-products formation was achieved at 160 °C for 2 h. High temperature (220 °C) and short time (0.5 h) contributed to hydrolysis of xylooligosaccharide with high DP to yield 37.5 wt% xylooligosaccharide with DP from 2 to 6. Xylooligosaccharide obtained mainly consisted of xylan with branches according to the HSQC NMR analysis. Overall, the production of XOS with a high yield from food waste will facilitate the valorization of food waste in the biorefinery industry.

**Graphical Abstract:**

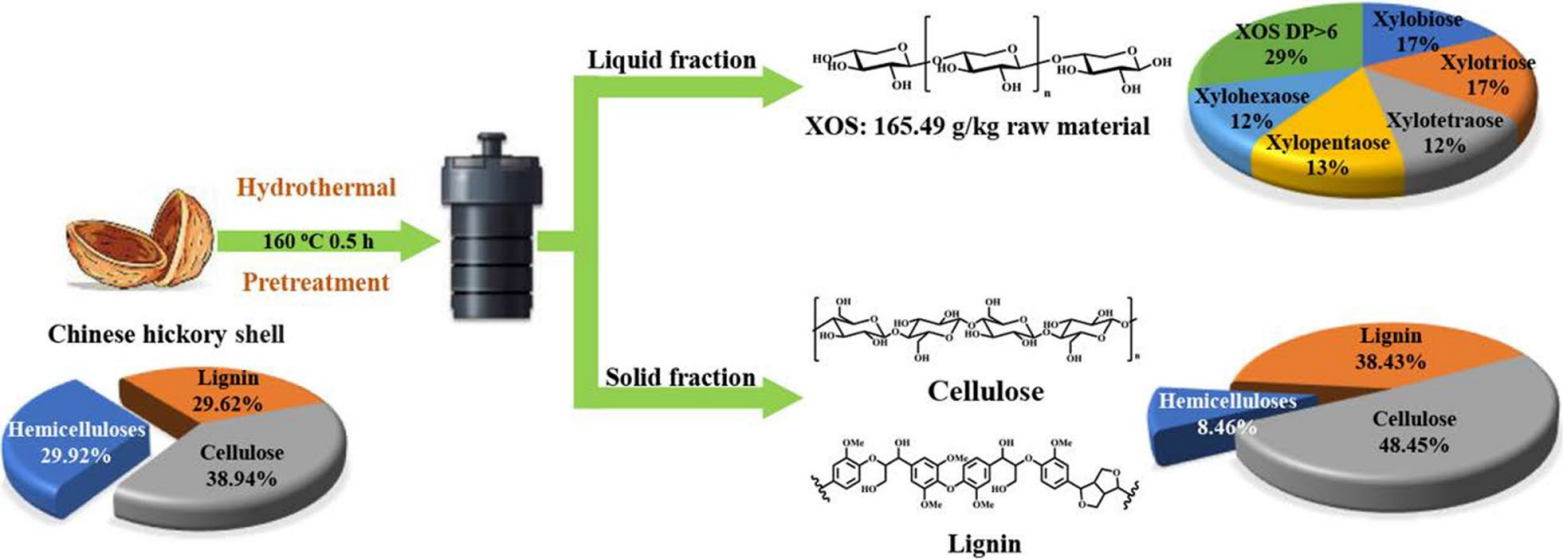

**Supplementary Information:**

The online version contains supplementary material available at 10.1186/s13068-021-02076-9.

## Introduction

Chinese hickory (*Carya Cathayensis*) is a famous nut due to its nutritional value. More than 15,000 ha of Chinese hickory trees are cultivated in the Zhejiang and Anhui provinces of China [1]. Recently, it has been reported that kernel, leaves, and green husk have abundant nutraceutical components [2]. However, the hickory shell is a by-product produced in large quantities after the cracking and shelling process. Furthermore, hickory shell as waste would pollute the environment [3]. As far as we know, there is no report that developing and using sustainable and green strategy is used for high value-added utilization of hickory shell. Therefore, how to utilize the hickory shell into value-added chemicals, materials, or biofuels is urgent and promising.

Similar to other biomass, hickory shell husk has high hemicelluloses, cellulose, and lignin contents. However, the differences in the structure and reactivity of these major components make the fractionation and utilization of hickory shell [4, 5]. In general, cellulose can be hydrolyzed to fermentable sugar for further biofuel production. At the same time, hemicelluloses were used as feedstock to produce gels, films, coating, and prebiotics in the biomedical, food, and pharmaceutical fields [6–8]. However, it also has high biomass recalcitrance, which results in the difficult utilization of lignocellulose [9]. Consequently, lignocellulosic biomass pretreatment is a prerequisite step for fractionation and utilization of hickory shell to overcome this challenge. Recently, various methods, including ultrasonic, enzymatic, microwave, hydrothermal, and a combination of these methods, have been applied to lignocellulosic biomass for fractionation of soluble components [10, 11]. According to previous studies, hydrothermal pretreatment is considered the economic and sustainable strategy that can promote reducing sugar and simultaneously producing value-added biochemicals, including hemicelluloses, oligosaccharides, and furfural [12–14]. Besides, there are no acid, alkali, organic solvents, and catalysts introduced during hydrothermal pretreatment, which is in accord with the principle of Green Chemistry [15].

During hydrothermal pretreatment, value-added chemicals, such as xylooligosaccharides (XOS) derived from hemicelluloses, can be produced [16]. Recently, the residues after pretreatment have been extensive investigations, but the lignocellulose is still underutilized to prepare XOS [17, 18]. Therefore, exploiting waste by industries to produce XOS is very promising from the perspective of economics and environment. Recent researches showed that XOS are non-digestible saccharides with prebiotic action [19–21]. Generally, sugarcane bagasse and corncob are used as feedstocks to prepare XOS [6, 22]. In addition, other lignocellulosic biomass, such as bamboo, brewery residues, also can produce XOS [13, 14, 16]. Hickory shell waste from Chinese hickory is the part of the volume of lignocellulosic biomass available and consists of ca. 30% hemicellulose. However, the production of XOS from this waste has not been reported until now.

Based on the reported research, acid, alkaline, and hydrothermal pretreatment are the main strategies for the fractionation of hemicelluloses and the production of XOS [6, 22]. Among these methodologies, hydrothermal pretreatment was gaining popularity relative and was considered an economically feasible method to produce significant amounts of XOS with a low degree of polymerization (DP) and inhibit the depolymerization of XOS [23, 24]. In the industry, the XOS with DP between 2 and 20 obtained only from hydrothermal treatment can be suitable for prebiotic applications [25]. Besides, hydrothermal pretreatment can catalyze the transformation of hemicelluloses during the reaction, while cellulose and lignin would not be degraded [26].

According to the lignocellulosic composition and the hydrothermal conditions in the production of XOS, the yield and DP of XOS are different. For example, 73.5 wt% of the xylan can be released from chestnut when the hydrothermal pretreatment was 180 °C, and the DP of XOS was related low (4.43) [24], while 45 wt% hemicelluloses of hazelnut shell were converted into XOS with DP ranging from 2 to 6 [27]. When the hydrothermal pretreatment temperature further increased, the DP of XOS produced from peanut shell and corn stover ranged from 2 to 17 and 6 to 15, respectively [28]. Although XOS can be obtained from various biomass, undesirable compounds, such as furfural, 5-hydroxymethylfurfural (HMF), and water-soluble lignin, were also produced during the hydrothermal pretreatment [6]. Therefore, how to produce XOS with a high yield from the hickory shell via hydrothermal pretreatment and avoid the formation of toxic products is much more significant and urgent.

Based on the above considerations, this research aims to exploit the hydrothermal pretreatment for the production of XOS from the hickory shell for the revelation of this industrial waste. Meanwhile, the constitutional changes of the hickory shell by characterization of the dissolved components (XOS, monosaccharides, and water-soluble lignin) were systemically evaluated to understand the fractionation and degradation of the hemicelluloses in hickory shell during the hydrothermal pretreatment. The effects of hydrothermal pretreatment on the transformation of hemicelluloses were investigated by high-performance anion-exchange liquid chromatography (HAPEC), high-performance liquid chromatography (HPLC), as well as two-dimension heteronuclear single quantum coherence (2D-HSQC) to reveal the XOS formation from the hickory shell. Besides, the characteristics of soluble products were systemically elucidated. This research would provide valuable results in the commercial exploitation of hickory shell for industrial production of biochemicals from hickory shell waste.

## Results and discussion

In this work, the Chinese hickory shell was pretreated with deionized water at solid/liquid of 1:10 and pretreatment temperatures of 140, 160, 180, 200, and 220 °C for 0.5, 1, 2, and 4 h, respectively. In general, hydrothermal pretreatment can deconstruct lignocellulose and break the biomass recalcitrance, which fractionates the lignocellulose into water-insoluble solid and water-soluble liquid fractions [12, 16]. As shown in Additional file 1: Fig. S1, the hickory shell without pretreatment appeared a smooth and regular surface. By contrast, the surface of the pretreated substrate began to appear slight cracks and expose the internal structure of the lignocellulosic residue with the increase in temperature and treatment duration. Furthermore, it was found that many holes and loose structure appeared on the surface of the substrate pretreated at 200 °C. The reason for this was mainly that the degradation of a considerable amount of hemicelluloses during the pretreatment opened up macropores. Besides, due to the hemicelluloses removal, the CrI value of the pretreated substrates increased (Additional file 1: Fig. S2). The above data indicate that hydrothermal pretreatment did reduce the recalcitrance of the Chinese hickory shell.

Water-insoluble solid mainly consisted of cellulose and lignin. Due to the reduction of biomass recalcitrance, cellulose can be readily hydrolyzed into glucose, and the left lignin can further be used to produce lignin-based resin. The water-soluble liquid fraction was mainly composed of hemicelluloses-based monosaccharides and XOS. Meanwhile, by-products were also generated during the hydrothermal process, especially in harsh reaction conditions. As a result, the liquid fraction is very complicated. Consequently, it should qualitatively and quantitatively analyze the liquid fraction to promote the utilization of Chinese hickory shell.

### The yield of the solid and dried liquid fraction

Since complicated by-products would be formed, which was terrible for utilizing hemicelluloses, the ultimate goal is that most of the hemicelluloses in lignocellulose were released and converted into XOS, but not further into furfural, levulinic acid, formic acid, and other by-products. Herein, the yield of solid and dried liquid fraction after pretreatment was investigated.

**Fig. 1:**
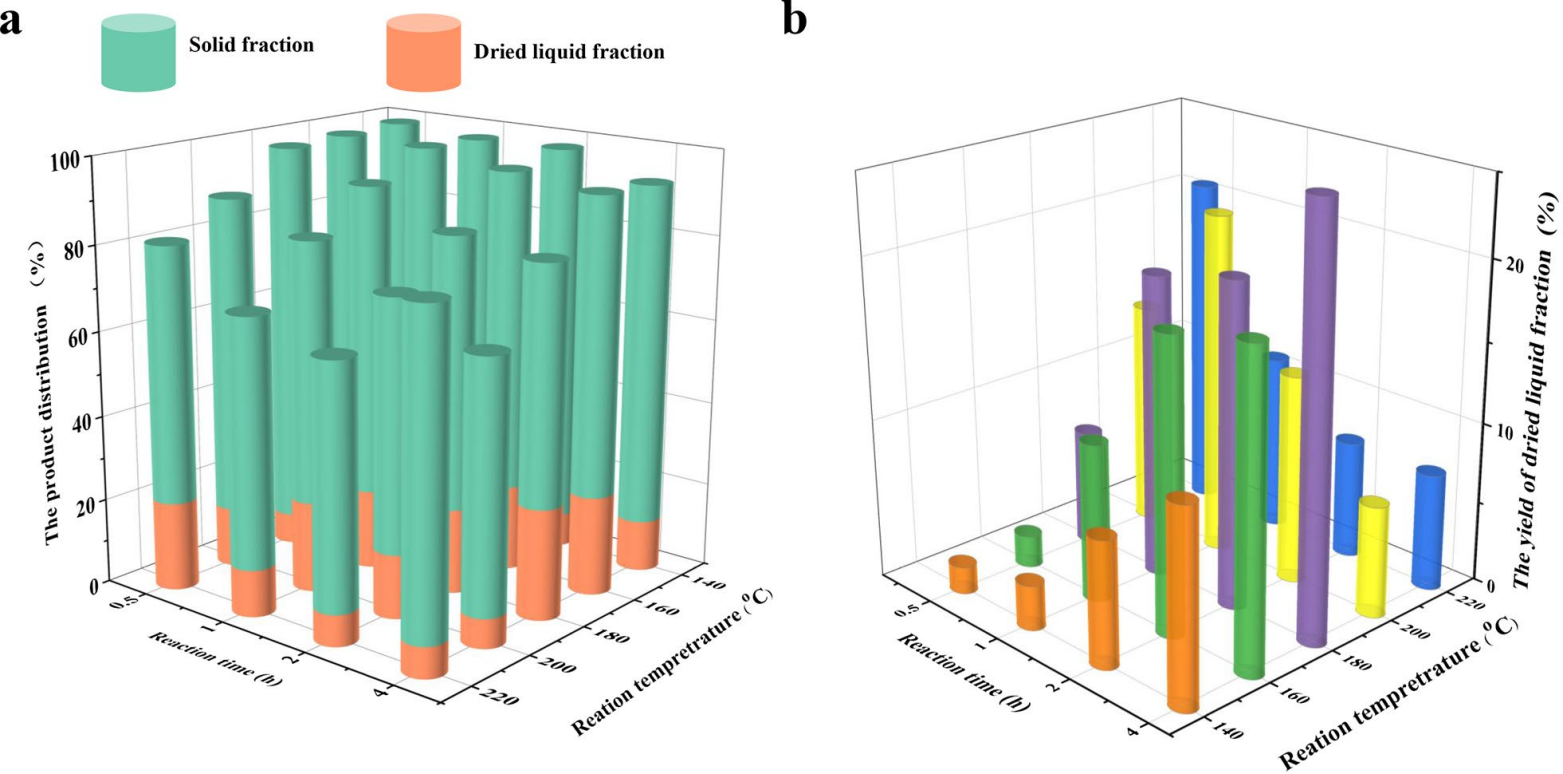
**a** The total yield of solid fraction and dried liquid fraction after hydrothermal pretreatment, **b** the yield of dried liquid fraction after hydrothermal pretreatment

As shown in Fig. 1a and Additional file 1: Table S1, the reaction conditions strongly affect the total yield of solid and dried liquid fractions. The higher the reaction temperature was, the longer reaction time was, the total yield was much declined from 98.73 to 63.93%, which mainly was ascribed to the factor that hemicelluloses and amorphous cellulose were severely degraded into by-products (furfural, HMF, levulinic acid, lactic acid, acetic acid, and formic acid) under harsh reaction conditions [12]. When the pretreatment condition was further harsher (temperature 220 °C, time 4 h), the by-products would be condensed to form humin and precipitate in the solid fraction, improving the yield of the solid fraction.

Generally, the hydrolysis products in the liquid fraction were mainly XOS and monosaccharides. Figure 1b and Additional file 1: Table S1 show the yields of dried liquid fraction as the pretreatment temperature and time increased during the hydrothermal process. Under mild temperature (≤ 180 °C), a long time would promote the yield of dried liquid fraction. Meanwhile, the water-soluble components would be further degraded into small molecules under harsh reaction conditions (≥ 180 °C), decreasing the dried liquid fraction yield. At 200 °C for 1 h, the yield of the dried liquid fraction can be up to 21.27 wt%. However, under high temperature, part lignin in the lignocellulose also would be dissociated into the water, which improved the yield of dried liquid fraction. On the other hand, lignin introduced XOS-rich liquid fraction will increase the difficulty of separation and purification of XOS. Therefore, preventing dissociation of lignin and degradation of XOS and improving the release of hemicelluloses is a challenge during biomass pretreatment.

### Composition analysis of original and pretreated Chinese hickory shell

**Fig. 2:**
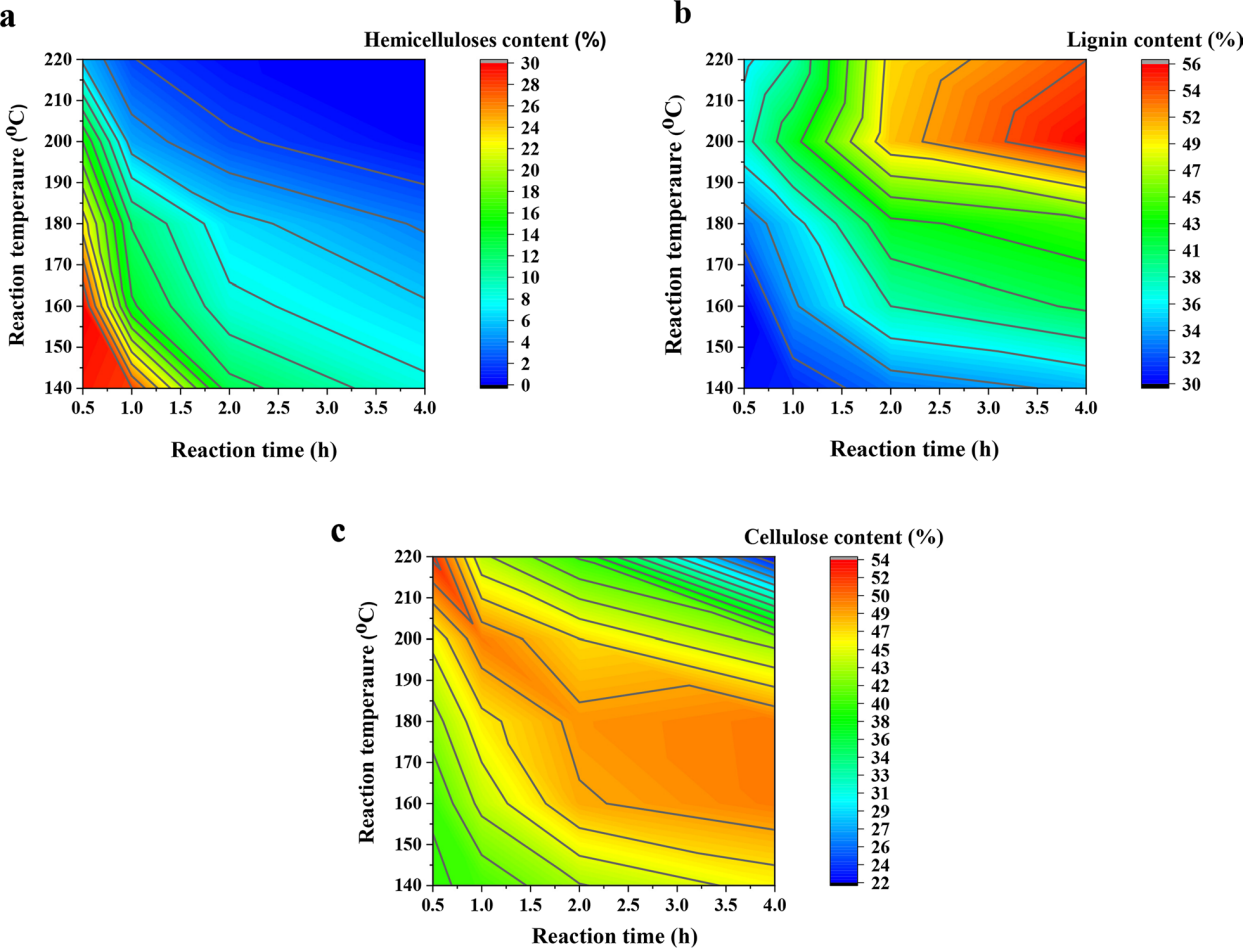
Chemical compositions of the pretreated substrates, **a** hemicelluloses content, **b** lignin content, **c** cellulose content

To understand the mechanism of hemicelluloses release and lignin dissociation during hydrothermal pretreatment, the chemical composition of the raw and pretreated Chinese hickory shell was determined by the NREL standard process [29]. Figure 2 and Additional file 1: Table S2 show the chemical composition and recovery yield of the shell with different pretreatment conditions. The chemical composition of the raw Chinese hickory shell was 38.94% cellulose, 29.92% hemicelluloses, and 29.62% lignin. As compared to lignin and cellulose, hemicelluloses are much more readily released and degraded from lignocellulose. After pretreatment, the contents of hemicelluloses in the pretreated shell rapidly decreased, especially in severe conditions. This was attributed to the fact that the water autoionization can form the hydronium ions (H_3_O^+^), resulting in hemicelluloses' degradation by selective cleavage of glycosidic linkages [30]. Simultaneously O-acetyl group also was hydrolyzed to form acetic acid, which further promoted the degradation of hemicelluloses [31].

As shown in Fig. 2a, high temperature or long time can be conducive to release most hemicelluloses from the lignocellulosic shell, which indicated hydrothermal pretreatment could efficiently remove hemicelluloses from plant cells. Unlike hemicelluloses, cellulose content firstly increased from 38.94% to ca. 50% due to the removal of hemicelluloses. While further elevated temperature or time, cellulose content sharply decreased, suggesting that some amorphous cellulose was degraded into glucose, HMF, and other chemicals under harsh conditions [7, 8]. Besides, it was observed that the content of lignin increased as the temperature and time elevated. The reason for this increase is that most hemicelluloses and part cellulose were degraded under harsh pretreatment conditions [32]. Notably, the lignin recovery in the pretreated shell was over 100% under the severe condition as shown in Additional file 1: Table S2, suggesting that condensation between carbohydrate-derived products and lignin led to the accumulation of pseudo-lignin at harsh conditions, which improve the content of lignin [30]. The above results indicated that hydrothermal pretreatment could efficiently fractionate the lignocellulose and break biomass recalcitrance, especially when most hemicelluloses was released into liquid, favoring the production of XOS.

### Monosaccharide and XOS analysis of the dried liquid fractions

During hydrothermal pretreatment, the release of hemicelluloses was triggered by the hydronium ion formed in situ by autoionization of water and further heightened by organic acids produced from the degradation of hemicelluloses [33]. Generally, the liquid fraction generated from hydrothermal pretreatment mainly consisted of the products degraded from hemicelluloses, including oligosaccharides, monosaccharides, and furfural [6]. During the hydrothermal process, the hemicelluloses were stepwisely converted into oligosaccharides via hydrolysis of glycosidic bonds and further degraded into monosaccharides, which would also be reacted to form by-products via dehydration reaction [34]. This work tried to produce XOS with a high yield and prevent further degradation via optimized reaction conditions.

**Fig. 3:**
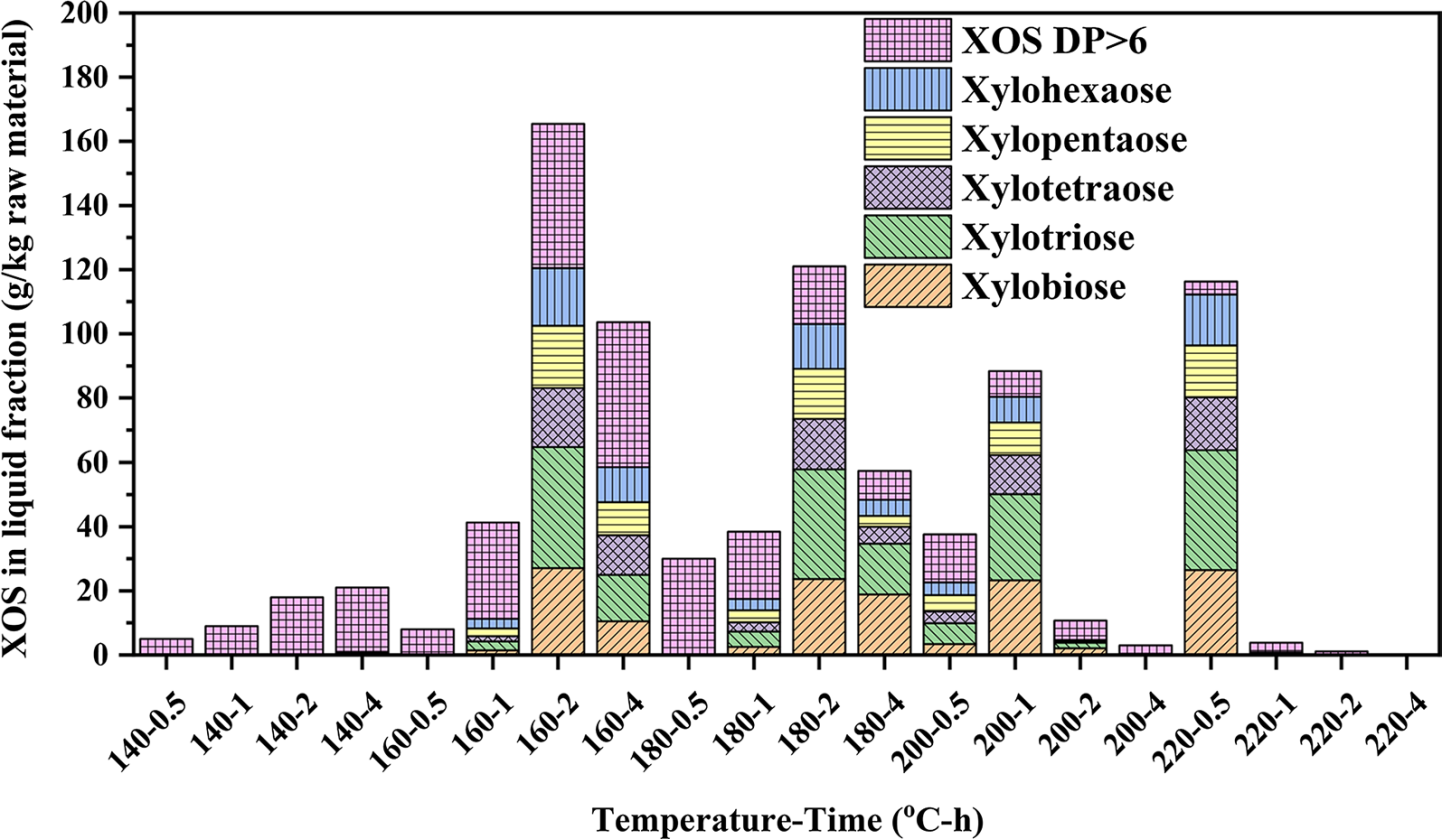
Production of XOS from Chinese hickory shell after the hydrothermal pretreatment

In this study, the liquid fraction obtained from hydrothermal pretreatment was rich in XOS. Therefore, the amount of XOS with different DPs was also measured. As shown in Fig. 3, the effects of the reaction temperature and time in the hydrothermal pretreatment on the yield and composition of XOS from Chinese hickory shell were compared. As can be observed, the total yield of XOS increased slowly as pretreatment time was prolonged at 140 °C. When pretreatment temperature elevated to 160 °C, the yield increased sharply to the maximum value (165.49 g/kg raw material) when time prolonged to 2 h. Still, too long time would further degrade XOS into monosaccharides and other by-products, which confirmed that properly prolonging pretreatment time was necessary for the production of XOS with a high yield at 160 °C during the hydrothermal process. As expected, when the pretreatment temperature was further increased to 220 °C, the XOS yield constantly decreased, especially prolonging time to 4 h at 220 °C, almost of XOS was degraded into xylose or other products, which was attributed to the factor that high temperature decreased the viscosity of the mixture and simultaneously increased the ionic mobility resulting in releasing more hydronium ions for degradation and dehydration of hemicelluloses into the xylose and other small organic molecules [35]. Besides, it was observed that the contents of high-DP XOS were high at mild conditions (low temperature and short time). While the condition became harsh, the proportion of XOS with DP ranging 2 to 6 increased due to the hydrolysis of XOS with high DP. In general, intensifying reaction condition (expressing the logR_0_ value) can improve the yield of XOS due to removal and hydrolysis of hemicelluloses (Additional file 1: Table S3); however, harsh reaction condition led to the extensive degradation of XOS, which decreased the yield of XOS. The maximum yield of XOS with DP ranging from 2 to 6 was 112.27 g/kg raw material at 220 °C for 0.5 h. Taken together, the optimum condition for the production of XOS with wide DP was at 160 °C for 2 h, while 220 °C and 0.5 h is the best condition for the preparation of XOS with DP ranging from 2 to 6.

**Fig. 4:**
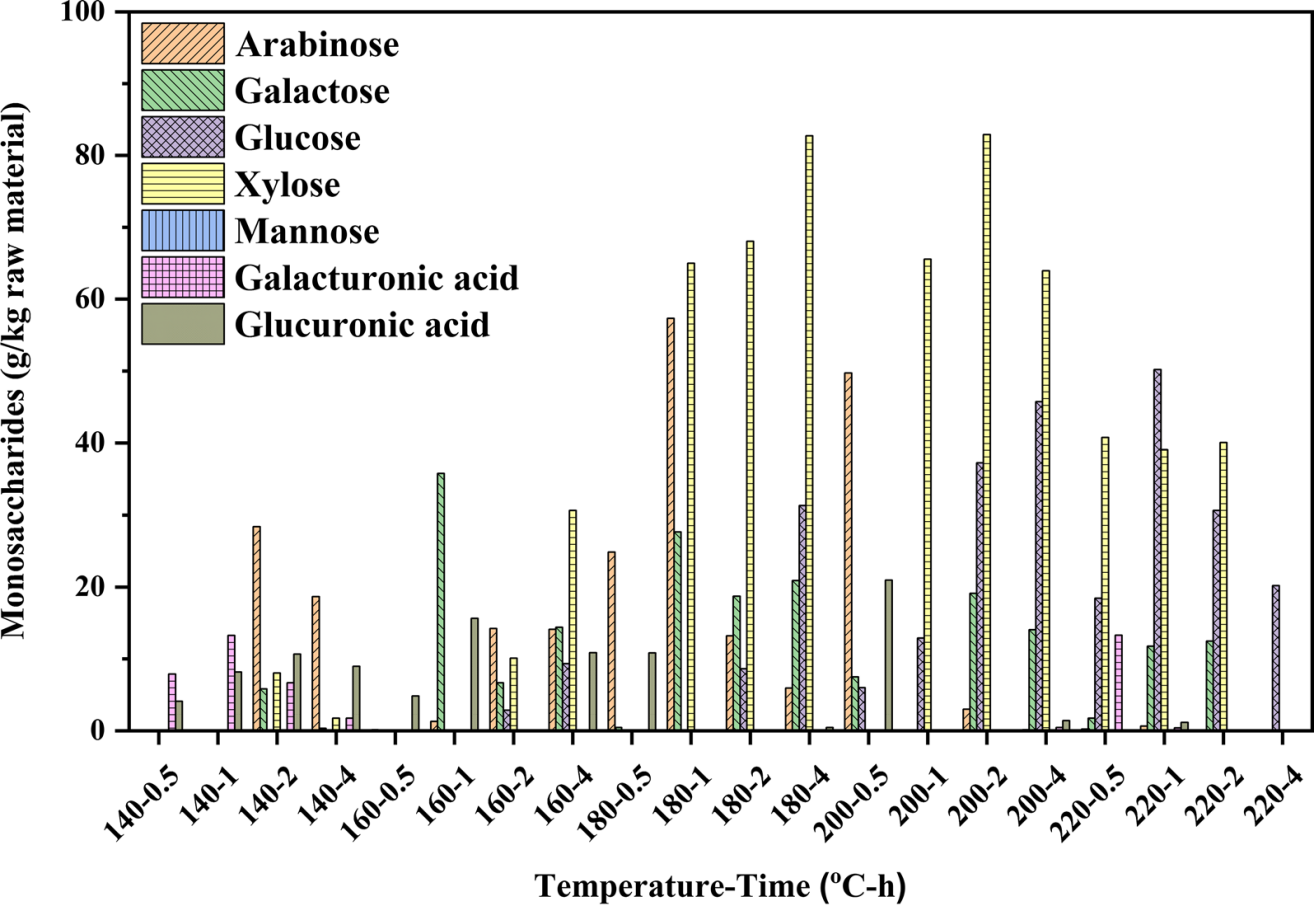
Production of monosaccharides from Chinese hickory shell after the hydrothermal pretreatment

Figure 4 also illustrates that the monosaccharide was obtained at various pretreatment conditions. Arabinose, galactose, glucose, xylose, mannose, galacturonic acid, and glucuronic acid were observed in the liquid fraction, and xylose was the primary product after hydrothermal pretreatment due to the high content of xylan in the raw material. Arabinose and galactose had a relatively high yield under mild reaction conditions. As the temperature increased and time prolonged, xylose was the primary product since the XOS obtained from hemicelluloses was further hydrolyzed into xylose under high temperature and a long time, which agreed with the quantitative analysis of XOS. At the same time, cellulose also was be degraded into glucose. In general, as shown in Fig. 4, high temperature and long time would promote the hydrolysis of hemicelluloses and cellulose into pentose and hexose. Still, the yield of pentose and hexose was lower than that of XOS under the best condition for the production of XOS.

### By-products distribution of the liquid fraction

Monosaccharides obtained from hemicelluloses during the hydrothermal process would be further degraded into furfural, HMF, and carboxylic acids (formic, acetic, levulinic, and lactic acids) [34]. Generally, the acetyl groups of hemicelluloses were cleaved to produce acetic acid under the hydrothermal process [7]. Meanwhile, the pentose was dehydrated into furfural and was further degraded into formic acid under acidic conditions, and hexoses (glucose, galactose, and mannose) can be transformed into HMF and further degraded into levulinic and formic acids [30].

**Fig. 5:**
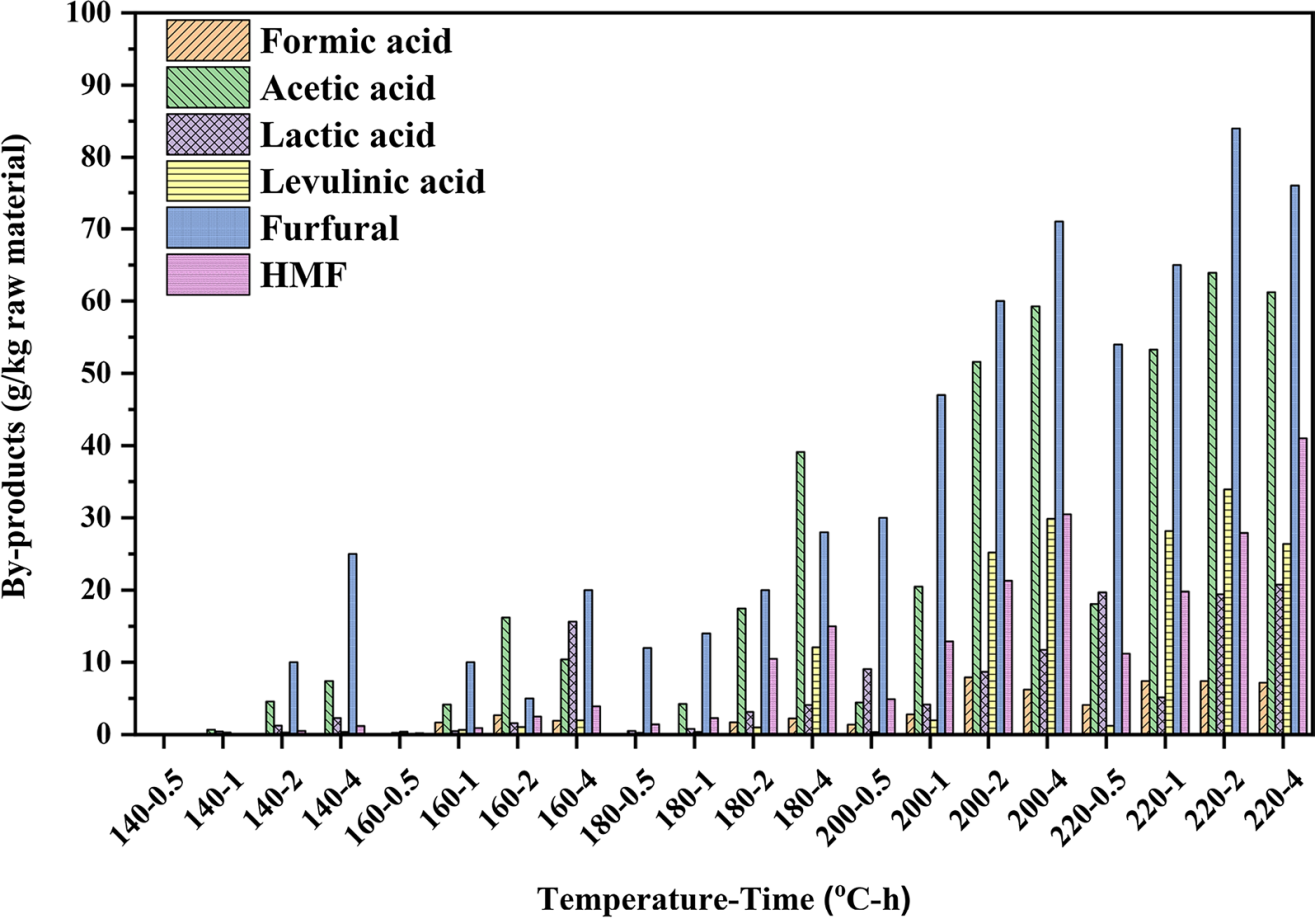
By-products in the liquid fraction after hydrothermal pretreatment

As shown in Fig. 5, furfural, HMF, formic acid, acetic acid, levulinic acid, and lactic acid were the main products in the liquid fraction. The yield of acetic acid (63.91 g/kg raw material) and furfural (84.73 g/kg raw material) was much higher than that of other by-products (< 40 g/kg raw material) at the harsh pretreatment condition. However, with the maximum yield of XOS with wide DP obtained at 160 °C for 2 h, the yield of acetic acid was only 16.21 g/kg raw material, which can prevent the degradation of XOS. Besides, the low yield of acetic acid (18.06 g/kg raw material) also can be found in the best condition of the yield of XOS with DP ranging from 2 to 6. Interestingly, the formation of all by-products was strongly related to the pretreatment condition, that is, the yield of by-products increased as the temperature elevated and time prolonged, which were in good agreement with the previous reports [12, 34, 36]. As shown in Fig. 5, it was found that the hydrothermal process under the higher temperature for a long time favored the dehydration of pentose and hexose as compared to that formed at other mild reaction conditions. As compared to the maximum yield of XOS (165.49 g/kg raw material) at 160 °C for 2 h, the amount of the by-products in the liquid fraction was at a quite low level (the amount of formic acid, acetic acid, lactic acid, levulinic acid, furfural, and HMF was 2.70, 16.21, 1.59, 1.05, 5.01, and 2.50, respectively), which indicated hydrothermal pretreatment is an efficient strategy with fewer by-products for lignocellulosic Chinese hickory shell to prepare XOS with high yield.

### Structural analysis of XOS in the liquid fraction

To further obtain the detailed information on the various structures features and changes of the dried liquid fraction under the 140, 160, and 220 °C for 2 h and retention of 0.5, 2, and 4 h at 160 °C, 2D-HSQC NMR was carried out to analyze the substructures and chemical compositions. The cross-signals assignments in the HSQC spectra of carbohydrates and lignin are according to the previous reports and listed in Additional file 1: Table S4 [12, 34, 37]. In addition, typical structures of lignocellulose found in the liquid fraction are shown in Additional file 1: Fig. S2b to better analyze the NMR spectra of the liquid fraction.

The aliphatic regions of HSQC spectra of dried liquid fraction obtained after hydrothermal pretreatment are shown in Additional file 1: Fig. S3a. In this spectra region, the cross-signals of the methoxy group (-OCH_3_) were readily observed at *δ*_C_/*δ*_H_ 55.68/3.70. The cross-peaks of sugars from hemicelluloses were predominant in this region. Two obvious signals of xylan [X-I5 (C_5_–H_5_)] are located at *δ*_C_/*δ*_H_ 62.88/3.21 + 3.86; two non-reducing ends of xylan (X-NR5) were found at *δ*_C_/*δ*_H_ 65.50/3.03 + 3.60 [12, 38]. The other cross-peaks of xylan was also detected, and the X-I2 (C_2_–H_2_), X-I3 (C_3_–H_3_), X-I4 (C_3_–H_3_), X-R2 (C_2_–H_2_), X-R5 (C_5_–H_5_), X-NR3 (C_3_–H_3_), and X-NR4 (C_4_–H_4_) were observed at *δ*_C_/*δ*_H_ 72.39/3.04, 73.87/3.22, 75.35/3.48, 74.50/2.88, 58.63/3.51, 76.18/3.07, and 69.53/3.20 [38–40]. However, the chemical shifts of X-I2 and X-I4 overlapped with X-NR2 and X-R4, respectively. Besides, xylan with acetylation in the 2 (2-*O*-Ac-β-d-Xylp: C2/H2: *δ*_C_/*δ*_H_ 73.13/4.47) and 3 (3-*O*-Ac-β-d-Xylp: C2/H2, 74.61/4.75) positions were observed. As shown in Additional file 1: Fig. S3a, when the pretreatment time was 2 h, the intensity of the xylan signals increased as the temperature elevated from 140 to 160 °C. Further increasing temperature to 220 °C, most cross-signals became weak and even disappeared except methoxy group, glucan, and xylan. A similar tendency also can be observed as pretreatment time is prolonged.

Besides the above cross-signals, galactan (Gal2: *δ*_C_/*δ*_H_ 71.14/3.22 and Gal3: *δ*_C_/*δ*_H_ 73.10/3.35), mannan (3-O-Ac-β-d-Manp: *δ*_C_/*δ*_H_ 72.35/4.93 and Man3: *δ*_C_/*δ*_H_ 71.10/3.50), α-d-glucuronic acid (GlcA) (GlcA3: *δ*_C_/*δ*_H_ 69.50/3.62), and α-d-glucuronic acid (GlcA) (MeGlcA: *δ*_C_/*δ*_H_ 59.10/3.35) were also found in the aliphatic regions [13, 14, 38, 40, 41]. These signals only appeared at medium pretreatment conditions. This phenomenon was because hemicelluloses can be hydrolyzed into oligosaccharides under medium conditions and further be degraded into monosaccharides and by-products under harsh conditions. Glucan also showed its peaks at *δ*_C_/*δ*_H_ 72.76/2.87 (C-I2), 60.20/3.56 (C-I6), 70.20/3.20 (C-NR4), 61.10/3.39 (C-NR6), and 76.73/3.43 (C-I5, C-NR3, and C-NR5), respectively [38, 39, 42]. The cross-peaks of xylan and glucan became weak when the temperature decreased from 160 to 140 °C due to the restriction of low-temperature hydrothermal pretreatment, while high temperature also led to the further degradation of the oligosaccharides. A similar phenomenon was also observed when the pretreatment time was prolonged from 0.5 to 4 h at 160 °C.

As shown in Additional file 1: Fig. S3b, the signals of d-xylan, d-glucan, d-mannan, and d-galactan were predominant in the anomeric region, which was in agreement with the cross-peaks in the aliphatic region. Internal anomeric of the β-d-mannosyl [(1 → 4)-β-d-Manp)] was at *δ*_C_/*δ*_H_ 100.7/4.60, while signal of α-d-mannosyl [(1 → 6)-α-d-Manp)] was at δ_C_/δ_H_ 93.9/4.80 [40, 43]. Besides, the 3-O-Ac-β-d-Manp gave a signal at *δ*_C_/*δ*_H_ 99.90/4.60 [40]. Therefore, The XOS obtained from Chinese hickory shell via hydrothermal pretreatment also have some other oligosaccharides. As can be observed, the intensity of cross-peaks of the mannan sharply decreased as the temperature increased from 140 to 220 °C, due to the gradation of mannan. There is acetylated xylan structure observed at *δ*_C_/*δ*_H_ 99.31/4.49 and 100.97/4.32 [40]. Additional file 1: Fig. S3b showed that all acetyl groups were removed under high temperature, the signals of the reducing end of β-d-Xylp (*δ*_C_/*δ*_H_ 97.43/4.21) and α-d-Xylp (*δ*_C_/*δ*_H_ 92.4/4.85), and the [(1 → 4)-β-d-Xylp] (*δ*_C_/*δ*_H_ 101.61/4.26) of xylan backbone can be observed [40, 41]. Besides, some correlations of α-l-arabinofuranosyl were also observed at *δ*_C_/*δ*_H_ 106–109/4.7–5.10 under mild conditions [39, 44, 45]. Once pretreatment condition became harsh, the peaks of α-L-araban almost disappeared, which indicated α-l-araban was unstable in the hydrothermal process and quickly released and degraded. As shown in Additional file 1: Fig. S3b, the acetyl bonds were cleaved substantially as the condition became harsh.

Besides the signals of carbohydrates, water-soluble lignin also can be observed. As can be observed, the peaks from ferulates (FA), *p*-coumarates (PCE), guaiacyl (G), syringyl (S), and oxidized syringyl (S′) units are obvious in the HSQC spectra (Additional file 1: Fig. S4) [30, 37]. After hydrothermal treatment, the value of S/G of the liquid fraction decreased as the pretreatment severity increased, suggesting the S units were much easily degraded under hydrothermal process at the high severity, which was in agreement with the previous reports [12, 34, 36]. Besides the peaks of the typical lignin structure, some condensed signals for lignin were also observed under severe conditions. Although lignin can be dissociated from Chinese hickory shell and released into liquid fraction, the content is much less than that of XOS. Besides, it was also found that water-soluble lignin was rich in phenolic hydroxyl group, which gave water-soluble lignin good antioxidation. Therefore, there is reason to believe that XOS with some water-soluble lignin have much better performance in the field of prebiotics [46].

### Process mass balance

**Fig. 6:**
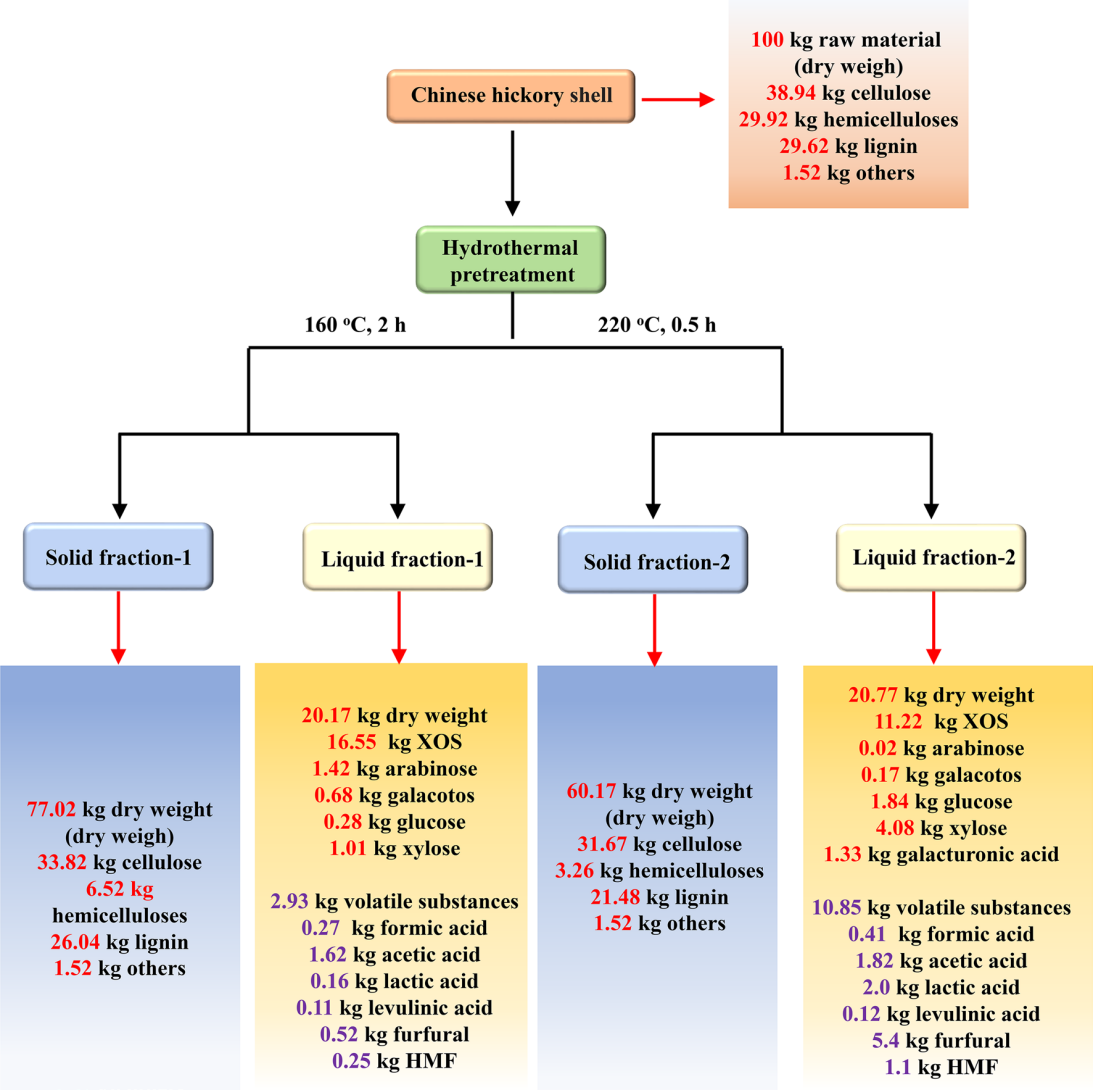
Mass balances during the hydrothermal pretreatment for two pretreatment conditions (160 °C, 2 h and 220 °C, 0.5 h)

In this work, the maximum yield of XOS with wide DP ranging from 2 to 6 was achieved at 160 °C for 2 h and 220 °C for 0.5 h with a relatively low yield of monosaccharide and by-products. Excessively prolonged time or increased temperature sharply decreased the yield of XOS and improved the amount of monosaccharides and by-products. Herein, a process mass balance of the hydrothermal pretreatment was developed for the two pretreatment conditions (160 °C for 2 h and 220 °C for 0.5 h). (Fig. 6). Process yields of products in the solid and liquid fractions were normalized to a common basis of 100 kg raw Chinese hickory shell as the starting material. After hydrothermal pretreatment, liquid and solid fractions were separated by filtration. Under medium condition (160 °C for 2 h), hemicelluloses were converted into XOS from lignocellulose and were limited to further degrade into monosaccharide and by-products, cellulose, and lignin still remain in the solid lignocellulose. In this condition, it was found that 16.55 kg XOS with wide DP can be obtained, while only a small amount of lignin was observed in the dried liquid fraction. Further increasing temperature to 220 °C, most hemicelluloses were removed from lignocellulose. Meanwhile, some lignin is also dissociated into the liquid fraction. Shorted time can prevent hydrolysis of oligosaccharides at high temperatures. Under this pretreatment condition (220 °C for 0.5 h), 11.22 kg XOS ranging from DP 2 to 6 from 100 kg raw material can be observed in the hydrothermal liquid. Besides, other more degraded products appeared in the liquid fraction due to the severe degradation of hemicellulose under high pretreatment temperature. These results showed that a relatively mild temperature and long time or a high temperature and short time was preferable for the production XOS.

## Conclusions

In this work, the Chinese hickory shell, a by-product of the food industry, was pretreated via the hydrothermal process. To understand the structural changes of hemicelluloses from lignocellulose to liquid fraction and obtain XOS with high yield, a detailed and systematic evaluation of solid and liquid fractions was conducted for the hydrothermal pretreatment of Chinese hickory shell at various temperatures and different times. The research showed that the pretreatment severity strongly affects the side chain constituents and backbone of xylan. With increased pretreatment temperature or time, the polysaccharides were hydrolyzed into oligomers and monomers and further degraded. Moderate temperature (160 °C) and relatively long time was preferred for the production XOS with wide DP, the maximum yield can be up to 165.5 g/kg raw material with a relatively low level of xylose and other degraded products, while relatively high temperature (220 °C) and short time (0.5 h) was in favor for preparation of XOS with DP from 2 to 6, the maximum yield was 112.2 g/kg raw material. The structural characteristics of the dried liquid fraction were also comprehensively elucidated by 2D-HSQC NMR. It was also observed that the XOS in the liquid fraction has a backbone of (1 → 4)-linked β-d-xylopyranosyl xylan decorated with branches. Meanwhile, lignin in the lignocellulose can be dissociated, degraded, and condensed. In short, the comprehensive evaluation of the liquid fraction during hydrothermal pretreatment of Chinese hickory shell will facilitate the valorization of food wastes in the biorefinery industry, especially the production of hemicellulose into XOS.

## Material and methods

### Materials

The raw Chinese hickory was harvested in 2019 from a local farm in Zhejiang Province, China. After drying, the kernel of Chinese hickory was removed, and the shell was ground to obtain a 20–80 mesh fraction for pretreatment. According to the NREL method, the raw Chinese hickory shell consisted of 38.94% cellulose, 29.92% hemicelluloses, 29.62% lignin, and 8.24% moisture [29].

### Hydrothermal pretreatment for production of XOS

The pretreatment experiments of Chinese hickory shell were carried out in a hydrothermal reactor with a stir bar. Typically, the reactor was charged with a suspension of 3.0 g Chinese hickory shell in 30 mL deionized water. Then the reactor was placed in an oil bath at the desired temperature by a PID temperature controller (model SX/A-1, Beijing Tianchen Electronic Company) under continuous stirring at 400 rpm within the desired time. After the reaction was completed, the reactor was placed in ice water, and the mixed liquor was separated into residual solid and filtrate by XOS-rich filtration via thoroughly washing with deionized water. Finally, the residual solid and XOS-rich filtrate were dried using a lyophilizer. Besides, the XOS-rich filtrate was also stored in a refrigerator for further analysis.

The intensity of the hydrothermal process, defined by the heating profiles, was measured according to the severity logR0 and was calculated using the following relation:

$$\log R_{0} = \log \left[ {\log \mathop \smallint \limits_{0}^{{t_{H} }} \exp \left( {\frac{{T\left( t \right) - T_{{{\text{REF}}}} }}{\omega }} \right) \cdot {\text{d}}t \,+\, t \cdot \exp \left( {\frac{{T\left( t \right) - T_{{{\text{REF}}}} }}{\omega }} \right)\, +\, \mathop \smallint \limits_{0}^{{t_{C} }} \exp \left( {\frac{{T\left( t \right) - T_{{{\text{REF}}}} }}{\omega }} \right) \cdot {\text{d}}t} \right]$$

where *t*_*H*_ (min) is the time needed to achieve the target temperature, *t*_*C*_ (min) is the time needed for the whole heating–cooling period, *t* (min) is the retention time, and *T*(*t*) represents the treatment temperature (°C). Calculations were made based on the values reported in the literature (*ω* and TREF are 14.75 and 100 °C, respectively) [47, 48].

### Characterization of original and residual Chinese hickory shell

The lignocellulosic compositions (%, w/w) of the original and residual Chinese hickory shell were determined according to the NREL standard analytical method [29]. The Chinese hickory shell before and after hydrothermal pretreatment was analyzed by scanning electron microscopy (SEM) and X-ray diffraction (XRD) according to the previous research [30].

### Characterization of the XOS-rich filtrate

According to our previous report, the amount of monosaccharides and XOS in the XOS-rich filtrate was measured by an HPAEC (Dionex ICS-3000, Thermal) with equipped a Carbopac PA-29 column [30]. The amounts of HMF, furfural, lactic acid, levulinic acid, acetic acid, and formic acid were analyzed by HPLC (Agilent 1200) according to the method reported [30]. In addition, the structure information of XOS-rich filtrate was measured by 2D-HSQC NMR [30]. NMR spectra were recorded with a Bruker AVIII 400 MHz spectrometer at 25 °C in DMSO-*d*_6_, and the pulse sequence “hsqcetgpsisp.2” was selected from Bruker Standard Pulse Library.

## Supplementary Information

**Additional file 1: Table S1.** The total yield of solid fraction and dried liquid fraction after hydrothermal pretreatment. **Table S2.** Chemical compositions and recovery of the raw and pretreated substrates. **Table S3.** The reaction condition, treatment severity (log R0), XOS from the hydrothermal treatment of Chinese hickory shell. **Table S4**. Assignments of 13C-1H cross-signals in the HSQC spectra of the liquid fractions obtained from Chinese hickory shell during hydrothermal treatment. **Figure S1.** SEM images of raw and the pretreated substrates under (a) different pretreatment temperatures and (b) different times. **Figure S2.** X-ray diffraction of raw and the pretreated substrates under (a) different pretreatment temperatures and (b) different times. **Figure S3.** (a) Aliphatic region and (b) anomeric region in the 2D HSQC. **Figure S4.** (a) Aromatic region in the 2D HSQC NMR spectra of the dried liquid after hydrothermal pretreatment, (b) Main typical substructures in lignocellulose of the dried liquid after hydrothermal pretreatment.

## Data Availability

All relevant data have been included in this published article and its Additional files.
