# Supplementary material for: Valorization of Chinese hickory shell as novel sources for the efficient production of xylooligosaccharides
Source: Biotechnol Biofuels. 2021 Nov 27;14:226. doi: 10.1186/s13068-021-02076-9 (PMC8626943; doi:10.1186/s13068-021-02076-9)
Supplement: Supplementary file 1 — Additional file 1: Table S1. The total yield of solid fraction and dried liquid fraction after hydrothermal pretreatment. Table S2. Chemical compositions and recovery of the raw and pretreated substrates. Table S3. The reaction condition, treatment severity (log R0), XOS from the hydrothermal treatment of Chinese hickory shell. Table S4. Assignments of 13C-1H cross-signals in the HSQC spectra of the liquid fractions obtained from Chinese hickory shell during hydrothermal treatment. Figure S1. SEM images of raw and the pretreated substrates under (a) different pretreatment temperatures and (b) different times. Figure S2. X-ray diffraction of raw and the pretreated substrates under (a) different pretreatment temperatures and (b) different times. Figure S3. (a) Aliphatic region and (b) anomeric region in the 2D HSQC. Figure S4. (a) Aromatic region in the 2D HSQC NMR spectra of the dried liquid after hydrothermal pretreatment, (b) Main typical substructures in lignocellulose of the dried liquid after hydrothermal pretreatment. [file 13068_2021_2076_MOESM1_ESM.docx]

**Supporting Information (SI)**

Table S1 The total yield of solid fraction and dried liquid fraction after hydrothermal pretreatment

| Pretreatment condition | | Dried liquid fraction  (%) | Solid fraction  (%) | Total yield  (%) |
| --- | --- | --- | --- | --- |
| Temperature (^o^C) | Time (h) |  |  |  |
| 140 | 0.5 | 1.71 | 97.02 | 98.73 |
|  | 1 | 2.79 | 94.53 | 97.32 |
|  | 2 | 7.83 | 89.92 | 97.74 |
|  | 4 | 12.00 | 80.36 | 92.36 |
| 160 | 0.5 | 1.99 | 96.02 | 98.01 |
|  | 1 | 9.88 | 87.93 | 97.80 |
|  | 2 | 20.17 | 75.02 | 95.19 |
|  | 4 | 23.31 | 69.73 | 93.04 |
| 180 | 0.5 | 7.17 | 90.32 | 97.50 |
|  | 1 | 18.74 | 72.87 | 91.60 |
|  | 2 | 20.00 | 63.77 | 83.77 |
|  | 4 | 25.97 | 55.46 | 81.43 |
| 200 | 0.5 | 14.00 | 74.43 | 88.43 |
|  | 1 | 21.27 | 61.02 | 82.29 |
|  | 2 | 15.00 | 58.58 | 73.58 |
|  | 4 | 6.92 | 58.17 | 65.09 |
| 220 | 0.5 | 20.77 | 60.17 | 80.94 |
|  | 1 | 11.00 | 57.99 | 68.99 |
|  | 2 | 7.37 | 56.56 | 63.93 |
|  | 4 | 7.27 | 72.85 | 80.12 |

Table S2 Chemical compositions and recovery of the raw and pretreated substrates

| Pretreatment condition | | Chemical compositions (%) | | | Recovery (%) | | |
| --- | --- | --- | --- | --- | --- | --- | --- |
| Temperature (^o^C) | Time (h) | Hemicelluloses | Lignin | Cellulose | Hemicelluloses | Lignin | Cellulose |
| - | - | 29.92 | 29.62 | 38.94 | 100.00 | 100.00 | 100.00 |
| 140 | 0.5 | 28.92 | 30.12 | 39.54 | 93.78 | 98.66 | 98.51 |
|  | 1 | 27.81 | 30.75 | 40.16 | 88.80 | 99.16 | 98.52 |
|  | 2 | 12.63 | 32.85 | 43.20 | 37.94 | 99.72 | 99.77 |
|  | 4 | 8.36 | 34.47 | 45.86 | 23.57 | 98.17 | 99.36 |
| 160 | 0.5 | 29.70 | 29.74 | 39.91 | 95.28 | 96.40 | 98.42 |
|  | 1 | 14.17 | 33.81 | 43.91 | 41.62 | 100.34 | 99.15 |
|  | 2 | 8.46 | 38.43 | 48.45 | 21.78 | 99.92 | 95.84 |
|  | 4 | 6.18 | 40.99 | 49.99 | 15.43 | 103.40 | 95.95 |
| 180 | 0.5 | 22.85 | 32.92 | 42.54 | 68.98 | 100.19 | 98.67 |
|  | 1 | 11.73 | 35.44 | 46.31 | 32.49 | 99.14 | 98.54 |
|  | 2 | 6.58 | 42.24 | 49.20 | 15.34 | 99.48 | 88.15 |
|  | 4 | 3.69 | 44.32 | 50.04 | 8.07 | 97.93 | 84.12 |
| 200 | 0.5 | 15.79 | 37.64 | 45.63 | 39.27 | 94.58 | 87.23 |
|  | 1 | 4.90 | 42.11 | 49.94 | 9.99 | 86.74 | 78.26 |
|  | 2 | 2.34 | 50.70 | 46.90 | 4.57 | 100.26 | 70.56 |
|  | 4 | 0.10 | 55.84 | 42.51 | 0.20 | 115.31 | 66.78 |
| 220 | 0.5 | 5.41 | 35.70 | 52.65 | 10.88 | 72.52 | 81.35 |
|  | 1 | 2.06 | 38.53 | 43.73 | 3.99 | 75.42 | 65.12 |
|  | 2 | 0.38 | 50.05 | 39.56 | 0.71 | 95.57 | 57.46 |
|  | 4 | 0.06 | 53.69 | 22.04 | 0.14 | 132.04 | 41.24 |

Note: The recovery (%) is based on the following equation (1)

$\text{Recovery}\left( \text{\%} \right)\text{=}\frac{\text{C}_{\text{Residual}}\text{×}\text{m}_{\text{Residual}}}{\text{C}_{\text{0}}\text{×}\text{m}_{\text{0}}}$ (1)

Where C_0_ and C_Residual_ are the chemical compositions in Chinese hickory shell before and after hydrothermal pretreatment, m_0_ and m_Residual_ are the mass of Chinese hickory shell before and after hydrothermal pretreatment.

Table S3 The reaction condition, treatment severity (log R0), XOS from the hydrothermal treatment of Chinese hickory shell

| Temperature (^o^C) | Time (h) | LogR_0_ | XOS  (g/kg raw material) |
| --- | --- | --- | --- |
| 140 | 0.5 | 0.90 | 5.12 |
|  | 1 | 1.20 | 9.03 |
|  | 2 | 1.50 | 18.24 |
|  | 4 | 1.80 | 20.97 |
| 160 | 0.5 | 1.50 | 7.89 |
|  | 1 | 1.80 | 41.34 |
|  | 2 | 2.10 | 165.49 |
|  | 4 | 2.40 | 103.62 |
| 180 | 0.5 | 2.10 | 30.31 |
|  | 1 | 2.40 | 38.45 |
|  | 2 | 2.70 | 121.06 |
|  | 4 | 3.00 | 57.41 |
| 200 | 0.5 | 2.69 | 37.63 |
|  | 1 | 3.00 | 88.34 |
|  | 2 | 3.30 | 10.74 |
|  | 4 | 3.60 | 3.05 |
| 220 | 0.5 | 3.29 | 116.27 |
|  | 1 | 3.59 | 3.84 |
|  | 2 | 3.90 | 1.14 |
|  | 4 | 4.20 | 0 |

Table S4. Assignments of ^13^C-^1^H cross-signals in the HSQC spectra of the liquid fractions obtained from Chinese hickory shell during hydrothermal treatment

| Lable | *δ*_C_/*δ*_H_ (ppm) | Assignments |
| --- | --- | --- |
| **–OCH_3_** | 55.68/3.72 | C-H in methoxyls |
| **X-I_2_** | 72.39/3.04 | (1→4)-β-D-Xyl*p* (C_2_/H_2_) of internal xylan |
| **X-I_3_** | 73.87/3.22 | (1→4)-β-D-Xyl*p* (C_3_/H_3_) of internal xylan |
| **X-I_4_** | 75.35/3.48 | (1→4)-β-D-Xyl*p* (C_4_/H_4_) of internal xylan |
| **X-I_5_** | 62.88/3.21,  62.88/3.86 | (1→4)*-*β-D-Xyl*p* (C_5_/H_5_) of internal xylan |
| **X-R_2_** | 74.50/2.88 | (1→4)-β-D-Xyl*p* (C_2_/H_2_) with reducing-end |
| **X-R_4_** | 75.35/3.48 | (1→4)-β-D-Xyl*p* (C_4_/H_4_) with reducing-end |
| **X-R_5_** | 58.63/3.51 | (1→4)-β-D-Xyl*p* (C_5_/H_5_) with reducing-end |
| **X-NR_2_** | 72.39/3.04 | (1→4)-β-D-Xyl*p* (C_2_/H_2_) with non-reducing-end |
| **X-NR_3_** | 76.18/3.07 | (1→4)-β-D-Xyl*p* (C_3_/H_3_) with non-reducing-end |
| **X-NR_4_** | 69.53/3.20 | (1→4)-β-D-Xyl*p* (C_4_/H_4_) with non-reducing-end |
| **X-NR_5_** | 65.50/3.03, 65.50/3.60 | (1→4)-β-D-Xyl*p* (C_5_/H_5_) with non-reducing-end |
| **MeGlcA** | 59.10/3.35 | 4-*O*-methyl-α-D-glucuronic acid (MeGlcA) |
| **GlcA_2_** | 67.60/3.56 | 4-*O*-methyl-α-D-glucuronic acid (C_2_/H_2_) |
| **GlcA_3_** | 69.50/3.62 | 4-*O*-methyl-α-D-glucuronic acid (C_3_/H_3_) |
| **X-MeGlcA_4_** | 76.66/3.59 | 4-*O*-methyl-α-D-glucuronic acid (C_4_/H_4_) linked to xylan at an *O*-2 position |
| **C-I_2_** | 72.76/2.87 | (1→4)-β-D-Glc*p* (C_2/_H_2_) of internal glucan |
| **C-I_5_** | 76.73/3.43 | (1→4)-β-D-Glc*p* (C_5_/H_5_) of internal glucan |
| **C-I_6_** | 60.20/3.56 | (1→4)-β-D-Glc*p* (C_6_/H_6_) of internal glucan |
| **C-NR_3_** | 76.73/3.43 | (1→4)*-*β-D-Glc*p* (C_3_/H_3_) with no-reducing-end glucan |
| **C-NR_4_** | 70.20/3.20 | (1→4)-β-D-Glc*p* (C_4_/H_4_) with no-reducing-end glucan |
| **C-NR_5_** | 76.73/3.43 | (1→4)*-*β-D-Glc*p* (C_3_/H_3_) with no-reducing-end glucan |
| **C-NR_6_** | 61.10/3.39 | (1→4)-β-D-Glc*p* (C_6_/H_6_) with no-reducing-end glucan |
| **Man_3_** | 71.10/3.50 | (1→4)-β-D-Man*p* (C_3_/H_3_) of Manan |
| **Gal_2_** | 71.14/3.22 | (1→4)-β-D-Gal*p* (C_2_/H_2_) of galactan |
| **Gal_3_** | 73.10/3.35 | (1→4)-β-D-Gal*p* (C_3_/H_3_) of galactan |
| **S_2,6_** | 103.70/6.60 | C_2,6_-H_2,6_ in syringyl units (S) |
| **S′_2,6_** | 106.70/7.21 | C_2,6_-H_2,6_ in oxidized (C*_α_*=O) S units (S′) |
| **G_2_** | 110.80/6.98 | C_2_-H_2_ in guaiacyl units (G) |
| **G_5_** | 114.70/6.71 | C_5_-H_5_ in guaiacyl units (G) |
| **G_6_** | 118.90/6.73 | C_6_-H_6_ in guaiacyl units (G) |


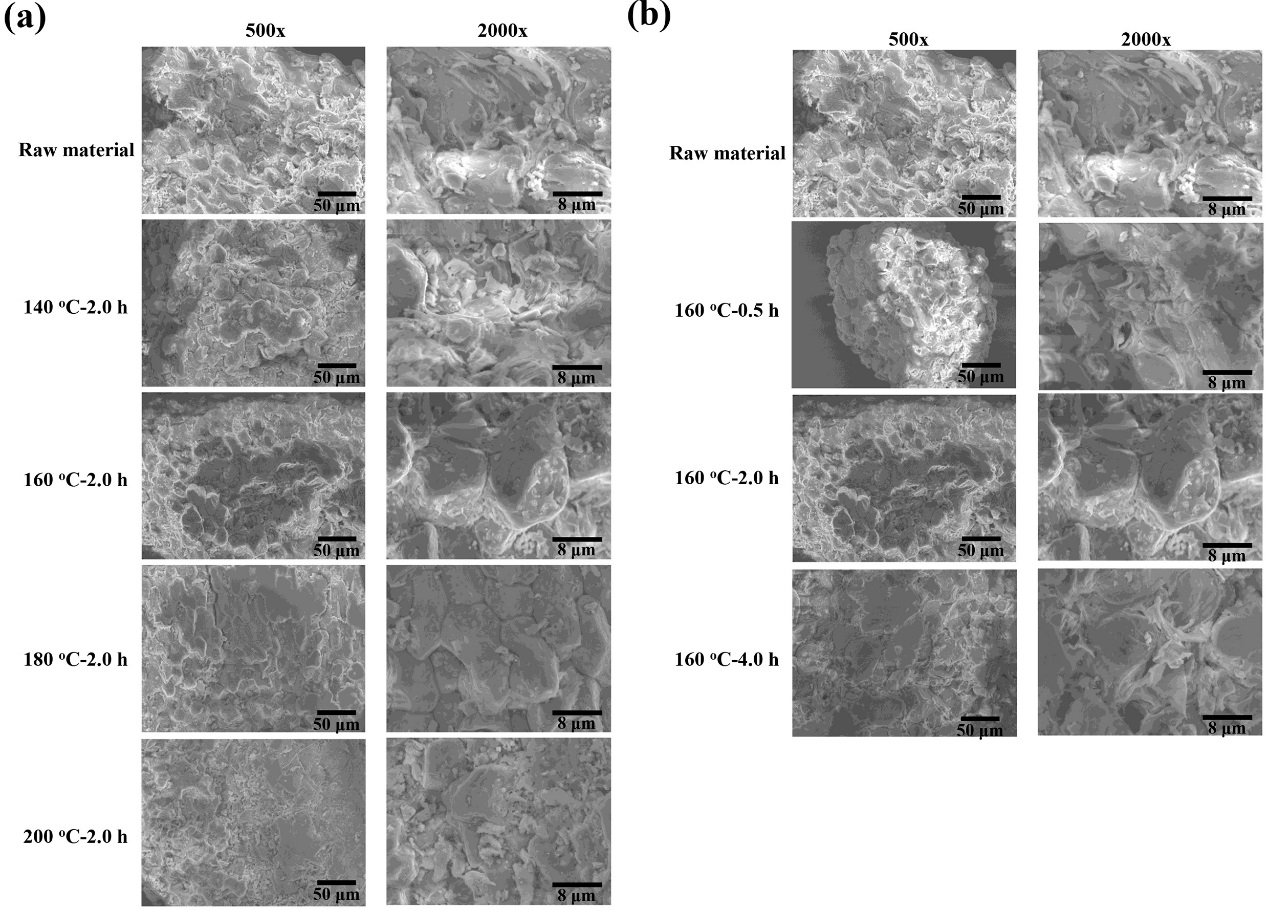


**Figure S1** SEM images of raw and the pretreated substrates under (a) different pretreatment temperatures and (b) different time.


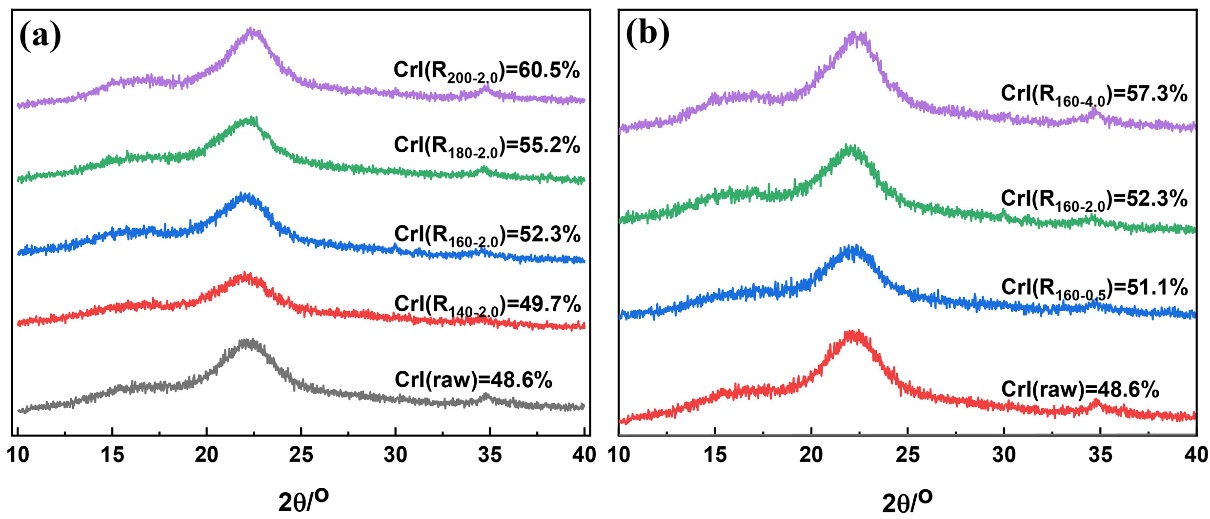


**Figure S2** X-ray diffraction of raw and the pretreated substrates under (a) different pretreatment temperatures and (b) different time.


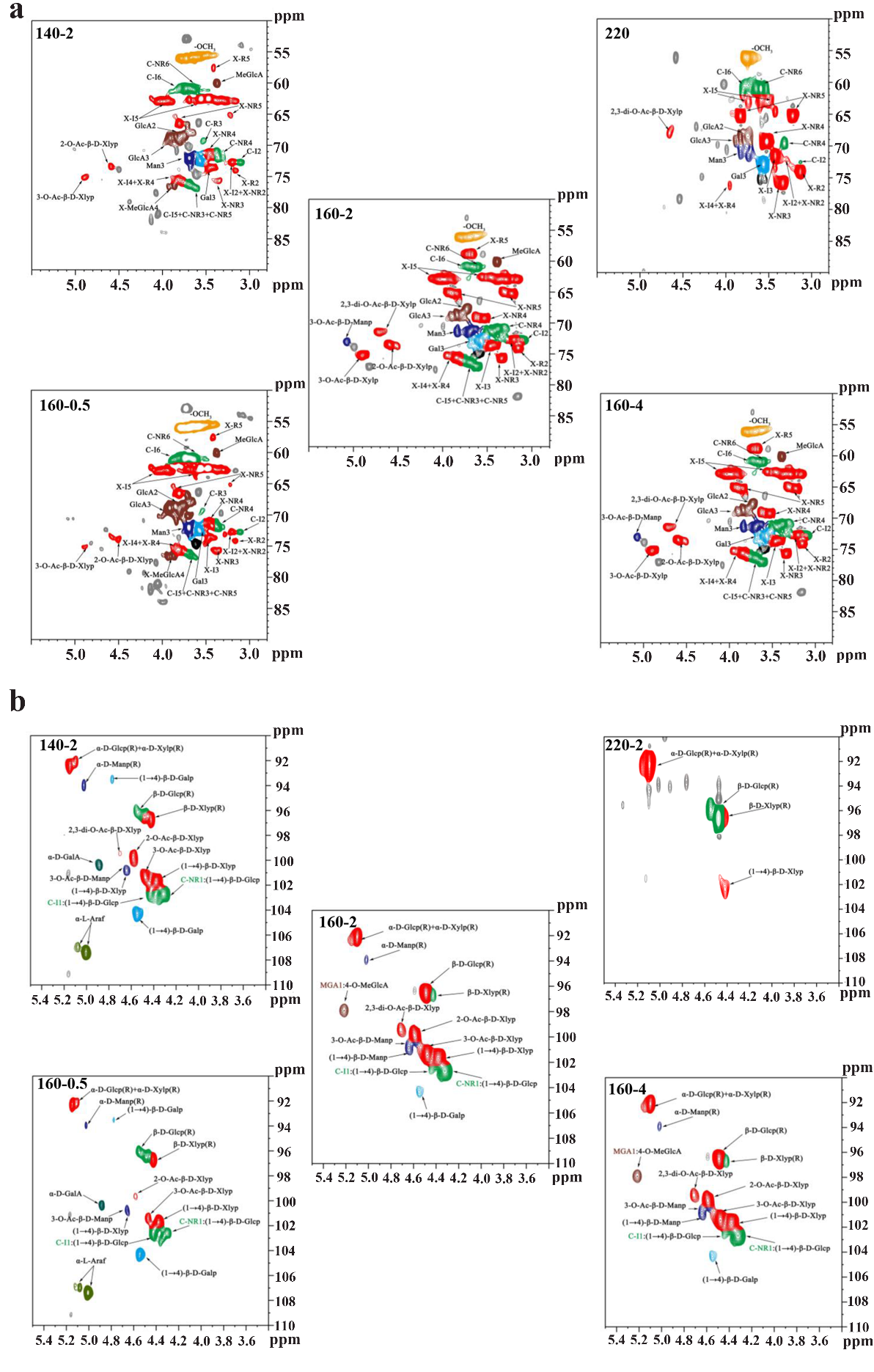


**Figure S3** (a) Aliphatic region and (b) anomerics region in the 2D‑HSQC
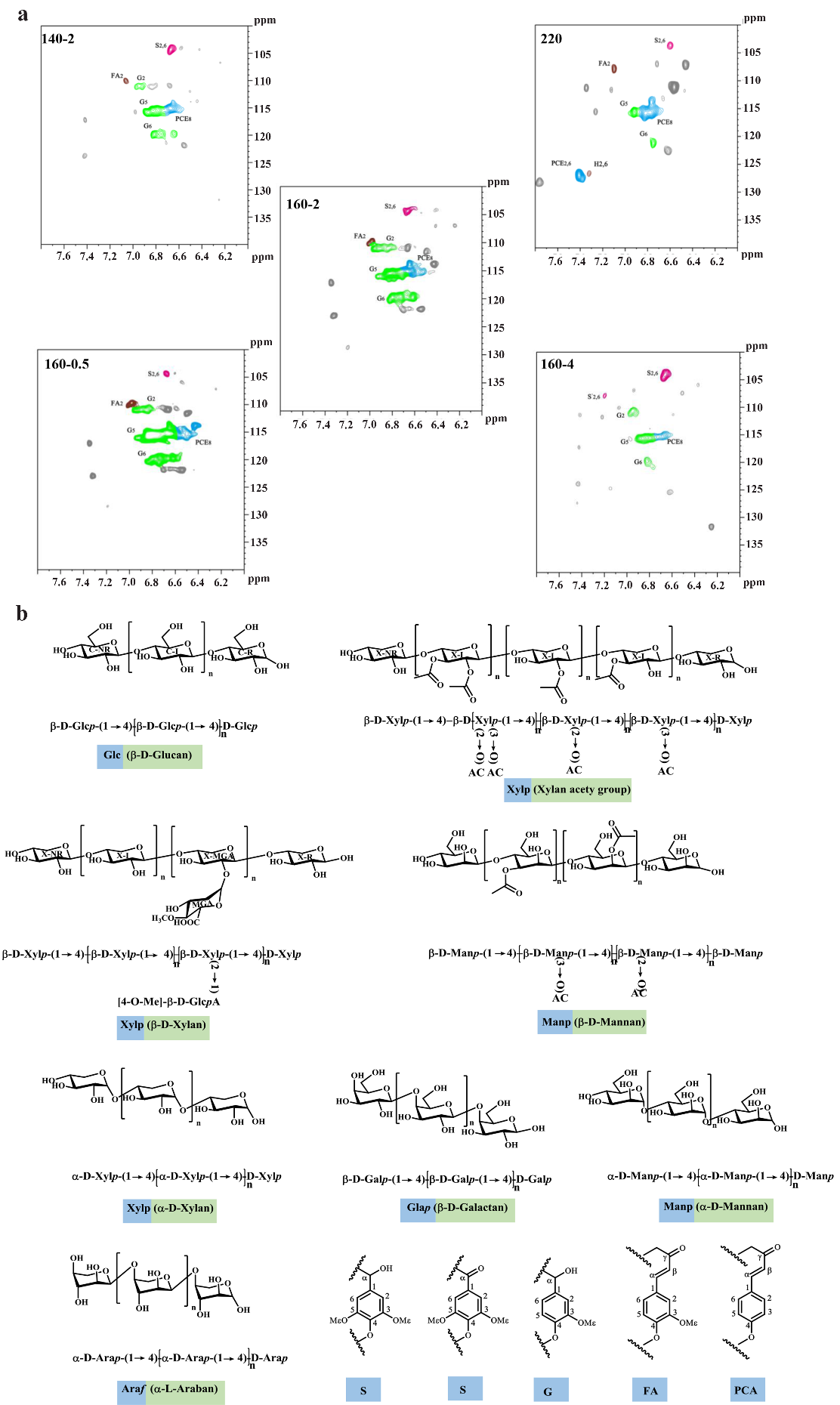


**Figure S4** (a)Aromatic region in the 2D‑HSQC NMR spectra of the dried liquid after hydrothermal pretreatment, (b) Main typical substructures in lignocellulose of the dried liquid after hydrothermal pretreatment
